# Preoperative Gamma-Glutamyltransferase Is Associated with Cancer-Specific Survival and Recurrence-Free Survival of Nonmetastatic Renal Cell Carcinoma with Venous Tumor Thrombus

**DOI:** 10.1155/2017/3142926

**Published:** 2017-01-11

**Authors:** Cheng Luo, Ben Xu, Yu Fan, Wei Yu, Qian Zhang, Jie Jin

**Affiliations:** Department of Urology, Peking University First Hospital and Institute of Urology, Peking University, National Urological Cancer Center, 8 Xishiku Street, Xicheng District, Beijing 100034, China

## Abstract

*Introduction*. To evaluate the prognostic significance of preoperative gamma-glutamyltransferase (GGT) on the subgroup of nonmetastatic renal cell carcinoma (RCC) with venous tumor thrombus.* Materials and Methods*. We retrospectively reviewed the institutional database and collected the medical data of 156 patients with nonmetastatic RCC with venous tumor thrombus between March 2004 and December 2014. Kaplan-Meier and Cox regression analyses were applied to determine the prognostic factors for cancer-specific survival (CSS) and recurrence-free survival (RFS).* Results*. The median value and optimal cutoff point of preoperative GGT were 23.0 and 37.5 IU/L, respectively. In the entire cohort, 67 (42.9%) patients experienced disease recurrence, and 46 (29.5%) patients died. Kaplan-Meier analysis revealed that the CSS and RFS rates were lower in patients with preoperative GGT ≥ 37.5 IU/L than in those with preoperative GGT < 37.5 IU/L. Multivariate Cox proportional hazard analysis demonstrated that high preoperative GGT was significantly associated with shorter CSS (hazard ratio [HR]: 2.115; 95% CI: 1.164–3.843; *p* = 0.014) and RFS (HR: 1.955; 95% CI: 1.166–3.276; *p* = 0.011), after adjusting other covariates.* Conclusions*. Preoperative GGT can serve as an independent prognostic biomarker of nonmetastatic RCC patients with venous tumor thrombus. Further prospective study is warranted to confirm our results.

## 1. Introduction

Currently, RCC represents the third most common malignancy of the urinary tract [1], but RCC with tumor tissue extending into the venous system is relatively rare [2]. Surgical management of RCC with venous tumor thrombus is one of the most technically challenging and complex urinary surgeries. Despite the development of multidisciplinary cooperation and operative skills, high rates of disease recurrence and cancer-specific mortality after surgical treatment remain an obvious concern in RCC patients with venous tumor thrombus [3]. The reported 5-year cancer-specific survival rates in this special subgroup ranged from 36.0% to 65.0% [4–7]. Considering the distinct heterogeneity of survival rates, several prognostic factors, including the tumor thrombus level, histological subtype, lymph node invasion, nuclear grade, were suggested to predict postoperative survival [8, 9]. Nevertheless, the reported prognosticators in previous studies were mainly pathological parameters, based on which the surgeons could not stratify the risks of poor prognosis preoperatively.

Recently, several preoperative laboratory biomarkers have been proposed to predict the prognosis of RCC patients; however, studies pertaining to the prognostic value of preoperative variables in RCC with venous tumor thrombus are limited. Haddad et al. investigated 166 RCC patients with tumor thrombus above the hepatic vein and revealed that an elevated level of preoperative serum alkaline phosphatase was associated with an increased risk of cancer-related death [10]. In addition, preoperative lactate dehydrogenase and C-reactive protein were demonstrated to be independent prognostic factors in another study with a relatively small sample [7].

GGT, which plays a pivotal role in cancer development, tumor progression, and anticancer-drug resistance [11], has been found to act as a significant prognostic biomarker in several cancer entities, such as hepatocellular carcinoma [12], metastatic breast cancer [13], esophageal squamous cell carcinoma [14], and ovarian cancer [15]. Specifically, there were two studies evaluating the prognostic impact of preoperative GGT in RCC patients. However, the final conclusions based on the results of multivariate Cox analyses were conflicting [16, 17]. Therefore, we conducted this study to assess the prognostic value of preoperative GGT in the subset of RCC patients with venous tumor thrombus.

## 2. Materials and Methods

### 2.1. Patient Selection

We retrospectively reviewed the medical records of 179 consecutive patients with RCC with venous tumor thrombus treated surgically between March 2004 and December 2014 at our institution. Patients who underwent radical nephrectomy with thrombectomy because of localized renal mass with venous tumor thrombus were included. The exclusion criteria were as follows: (1) patients with distant organ metastasis or other malignancies besides RCC; (2) those with hepatic diseases such as virus hepatitis, obstructive liver dysfunction, or drug-induced liver injury; (3) absence of preoperative laboratory test results. In addition, one patient was excluded because of death from postoperative ileus during hospitalization. Finally, 156 patients with nonmetastatic RCC with venous tumor thrombus were included for analysis. Approval of the institutional review board was obtained before initiating this study.

### 2.2. Preoperative Assessment

Preoperative abdominal contrast-enhanced computed tomography (CT) or magnetic resonance imaging was applied to identify the height of tumor thrombus. The grading of venous tumor thrombus was defined by the Neves classification [18]. Other examinations included routine laboratory tests as well as chest X ray/CT and bone scan for primary screening of distant metastasis. Additionally, brain imaging was considered if the patients complained of clinical symptoms. The serum GGT levels were examined by blood samples obtained within 3 days before the surgery. The measurements of GGT were performed in the institutional laboratory, which was recently accredited by the College of American Pathologists Laboratory Accreditation program.

### 2.3. Surgery and Postoperative Management

The surgical procedures were determined based on the tumor size, thrombus height, and discretion of the surgical team. Regional lymph node dissection was performed in patients with suspected nodal involvement on imaging or during the operation. Surgical specimens were examined by at least two experienced uropathologists. The subtype of RCC was identified according to the World Health Organization classification. Pathological tumor stage and grade were assigned according to the 2010 TNM classification and Fuhrman system. The follow-up after the surgical management was based on the scheduled visits, and the survival condition was checked by telephone in July 2016. The death reasons were confirmed with the death certificates of the hospital.

### 2.4. Statistical Analysis

The primary study endpoint was CSS, which was defined as from the date surgery to the date of death from RCC. The secondary endpoint was RFS, which was from the date of surgery to the date of disease recurrence. Receiver operating characteristic (ROC) curve analysis was performed to calculate the optimal cutoff point of preoperative GGT, based on which we could treat continuous GGT as a categorical variable and divide the entire cohort into two groups. The clinicopathological variables in different groups were compared using the Mann–Whitney *U* test or chi-squared test. The survival curves of CSS and RFS were depicted using the Kaplan-Meier method and were compared using the log-rank test. Univariate and multivariate Cox proportional hazard analyses were performed to establish the independent parameters for predicting the survival. Hazard ratios (HRs) were estimated from Cox proportional hazard analysis and were reported with corresponding 95% confidence intervals (CIs). The tumor thrombus level was excluded from multivariate analysis because of the intimate relationship with the pathological stage. All of the statistical analyses were conducted using SPSS 20.0 (IBM, Corp, Armonk, NY, USA). Statistically significant differences were considered when *p* value < 0.05.

## 3. Results

### 3.1. Basic Characteristics and Optimal Cutoff Point

**Figure 1:**
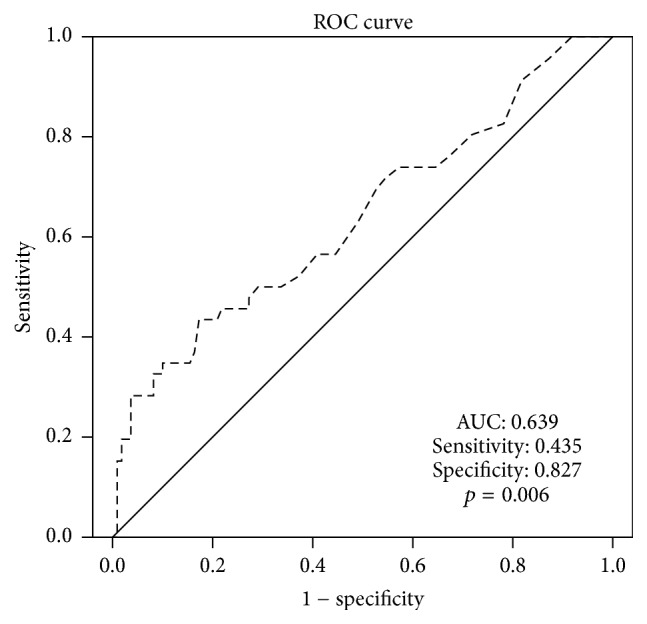
The ROC curve determining the optimal cutoff point of preoperative GGT.

The entire cohort consisted of 111 (71.2%) males and 45 (28.8%) females. The median (IQR) age at the time of surgery was 59.0 (51.0–66.0) years and the median (IQR) maximum tumor width was 8.5 (6.5–11.0) cm. According to the Neves classification, 85 (54.5%) patients were diagnosed with RV tumor thrombus, and 71 (45.5%) patients were diagnosed with IVC tumor thrombus. The median (IQR) value of the preoperative GGT was 23.0 IU/L. By performing ROC analysis, the optimal cutoff point of 37.5 IU/L was determined (Figure 1). Of the 156 patients, there were 117 (75.0%) patients with a preoperative GGT level greater than the cutoff point and 39 (25.0%) patients with a preoperative GGT level lower than the cutoff point. A high preoperative GGT was significantly associated with the IVC tumor thrombus level (*p* = 0.010), a high Fuhrman grade (*p* = 0.011), advanced pathological stage (*p* = 0.001), and the presence of sarcomatoid features (*p* = 0.010). The patient characteristics of the entire cohort and two groups according to the preoperative GGT are summarized in Table 1.

### 3.2. Survival Condition

**Figure 2:**
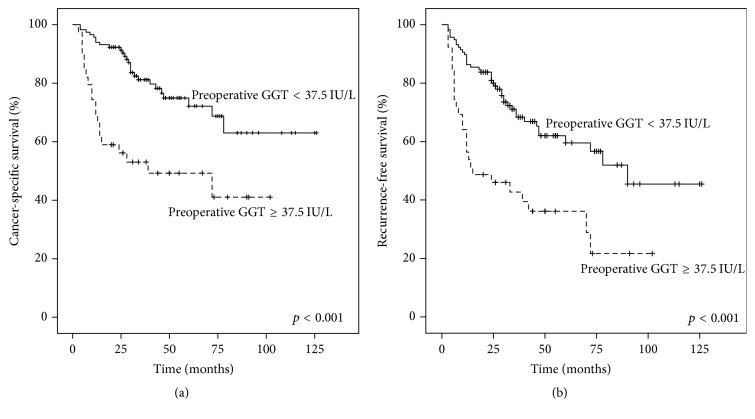
Kaplan-Meier curves of CSS and RFS stratified by preoperative GGT level. (a) Significantly worse CSS in high preoperative GGT group than in low-value group; (b) significantly worse RFS in high preoperative GGT group than in low-value group.

The median (range) follow-up duration was 34.0 (3.0–126.0) months. Among the 156 patients, 46 (29.5%) died from RCC. There were 26 (16.7%) patients with a preoperative GGT level greater than 37.5 IU/L and 20 (12.8%) patients with a preoperative GGT level lower than 37.5 IU/L. The 3-year and 5-year CSS rate were 81.0% and 72.0% in the high preoperative GGT group and 53.0% and 49.0% in their counterparts, respectively. Kaplan-Meier analysis demonstrated that the CSS rate was significantly different between the patients with and without elevated preoperative GGT levels (*p* < 0.001, Figure 2(a)). Additionally, 67 (42.9%) patients developed disease progression. The RFS rate was significantly lower in the high preoperative GGT group than in the low-value group (*p* < 0.001, Figure 2(b)).

### 3.3. Prognostic Value

Univariate Cox proportional hazard analysis identified that the presence of symptoms, high tumor thrombus level, large maximum tumor width, advanced pathological stage, high Fuhrman grade, and elevated preoperative GGT were poor prognostic factors for CSS. It was also illustrated that the tumor thrombus level, pathological stage, Fuhrman grade, and preoperative GGT were associated with RFS (Table 2). Further multivariate Cox model analysis revealed that preoperative GGT was a significant predictor of CSS (HR: 2.115; 95% CI: 1.164–3.843; *p* = 0.014) and RFS (HR: 1.955; 95% CI: 1.166–3.276; *p* = 0.011), independently of other included prognostic variables (Table 3).

## 4. Discussion

The present study evaluated the prognostic value of preoperative GGT in the patients with nonmetastatic RCC with venous tumor thrombus. We stratified the included patients into two groups according to the preoperative GGT level. Our results showed that patients with a high serum GGT level had a significantly worse prognosis than those with a low GGT level. After adjusting other prognostic variables, the preoperative GGT was determined to be an independent risk factor of CSS and PFS for this specially defined subgroup. Therefore, we recommended preoperative GGT as a potential predictor for clinicians to discriminate the patient survival before the treatment.

Preoperative GGT has been demonstrated to act as an independently prognostic biomarker in several cancer types. Regarding RCC, Sandock et al. evaluated the preoperative GGT level in RCC patients for the first time, and they found that the serum GGT was elevated in the most of the metastatic RCC cases compared with the localized RCC cases [19]. Furthermore, Hofbauer et al. conducted a survival analysis by investigating more than 900 consecutive RCC patients treated with nephrectomy. In this study, preoperative GGT was significantly associated with pathological T stage, lymph node stage, distant metastasis, Fuhrman grade, and the presence of tumor necrosis. Additionally, this parameter was determined as an independent risk factor for a worse oncologic outcome and could improve the predictive accuracy of previously reported prognostic models [16]. Nevertheless, a recent study involving a European cohort of nonmetastatic RCC patients failed to validate the prognostic significance of preoperative GGT [17]. A probable cause of this phenomenon was the composition of patients. Specifically, in the study conducted by Dalpiaz et al., the tumor stage of patients was mainly pathological T1 (67.4%), and the patients with metastasis were not included. Considering the uncertain predictive ability of GGT in low-risk patients, we deliberately selected the patients of locally advanced RCC with venous tumor thrombus to investigate the prognostic value of preoperative GGT.

In the subset of RCC with venous tumor thrombus, our results demonstrated that the elevation of the preoperative GGT level was significantly associated with advanced tumor stage and grade. Accumulating evidence has suggested that GGT is deregulated in cancer cells and could reflect tumor progression and aggressiveness [20]. Although GGT is limited to the brush border membrane of proximal tubules of the normal kidney tissues, it has been confirmed that GGT is expressed extensively on the membrane of RCC cells [21, 22]. As a membrane-bound enzyme, GGT plays a substantial role in sustaining the production of intracellular glutathione (GSH), which can function as an important antioxidant to protect cells from reactive oxygen compounds and free radicals. It was suggested that elevated GGT might help form a tumor microenvironment and protect the tumor cells from oxidative stress or cytotoxic drug [23]. On the other hand, GGT was reported to exert prooxidant effects under particular circumstances. The persistent oxidative stress contributed to the genomic instability and subsequent imbalance of cell proliferation and apoptosis, which were involved in tumor formation and progression [24]. Therefore, GGT could be treated as a biomarker of tumor aggressiveness by reflecting the extent of oxidative stress. In addition, it was reported that GGT could be induced by several inflammatory cytokines, including tumor necrosis factor alpha and interferon-alpha and interferon-beta [25, 26]. Thus, it was speculated that GGT was connected with tumor-associated inflammatory reactions and might act as an inflammatory biomarker to predict the prognosis of cancer patients. However, the exact direct mechanism of elevated GGT in carcinogenesis was finitely declared.

To the best of our knowledge, only two studies included nonmetastatic RCC patients with tumor thrombus to identify the risk factors of disease recurrence or progression [27, 28]. The reported preoperative prognostic factors to predict PFS are limited. The current study established that preoperative GGT is an independent prognostic parameter to predict PFS in the specially defined subgroup. Our findings are of interest from a clinical point of view. The serum GGT level is one of the most common liver function markers that is routinely tested on admission and is a low-cost and universally available preoperative prognosticator. Therefore, we recommend surgeons to consider the preoperative GGT level when distinguishing the patients' postoperative survival during preoperative evaluation or consultation. Several liver function markers, such as alkaline phosphatase and lactate dehydrogenase, have been reported to serve as prognostic biomarkers in the subset of RCC with venous tumor thrombus [7, 29]. Even so, the generalization of these parameters, including the serum GGT, needs further validation in prospective studies. To more comprehensively evaluate the survival of RCC patients with venous tumor thrombus preoperatively, other dependable biomarkers still need to be explored.

There are several limitations in this study. First, some unknown biases due to the inherent nature of the retrospective analysis were inevitable. Furthermore, the total sample size was small due to the rarity of RCC with venous tumor thrombus. However, the number of endpoint events observed was comparable to that of large-scale studies due to the high mortality of this subpopulation. Second, the serum GGT level could be impacted by various comorbidities of patients, and we could not exclude all of these influential factors. Nevertheless, all of the included patients underwent preoperative assessment by an anesthesiologist to evaluate the function of vital organs. Moreover, we strictly selected patients according to the predetermined exclusion criteria guaranteeing the relative homogeneity of the enrolled patients. Considering the listed defects, our results needed to be corroborated by future large-scale prospective studies.

## 5. Conclusions

Elevated preoperative GGT is associated with a poor prognosis of nonmetastatic RCC patients with venous tumor thrombus. The preoperative GGT level is an independent prognostic factor for clinical outcomes in the subset of RCC with tumor thrombus. A further prospective study with a larger sample size is needed to validate our results.

## Figures and Tables

**Table 1:** Clinicopathological features of the 156 patients according to preoperative GGT.

Variables	All patients, *n* (%) or median (IQR)	GGT, median (IQR), IU/L	*p* value	GGT ≥ 37.5 IU/L, *n* (%)	GGT < 37.5 IU/L, *n* (%)	*p* value
Number of patients	156	—	—	39	117	—
Median (IQR) age, years			0.474			0.711
>60	76 (48.7)	23.0 (16.0–38.0)		20 (51.3)	56 (47.9)	
<60	80 (51.3)	24.0 (16.3–37.0)		19 (48.7)	61 (52.1)	
Median (IQR) BMI, kg/m^2^			0.808			0.358
>25	45 (28.8)	23.0 (16.5–37.0)		36 (30.8)	9 (23.1)	
<25	111 (71.2)	23.0 (16.0–38.0)		81 (69.2)	30 (76.9)	
Gender			0.138			0.610
Male	111 (71.2)	24.0 (17.0–38.0)		29 (74.4)	82 (70.1)	
Female	45 (28.8)	21.0 (15.0–36.5)		10 (25.6)	35 (29.9)	
Symptom presentation			0.346			0.054
Yes	100 (64.1)	23.0 (17.0–39.0)		30 (76.9)	70 (59.8)	
No	56 (35.9)	23.0 (15.3–34.8)		9 (23.1)	47 (40.2)	
ASA			0.883			0.696
1 + 2	133 (85.3)	23.0 (16.0–38.0)		34 (87.2)	99 (84.6)	
3 + 4	23 (14.7)	26.0 (17.0–32.0)		5 (12.8)	18 (15.4)	
Tumor laterality			0.826			0.779
Right	89 (57.1)	23.0 (16.0–38.0)		23 (59.0)	66 (56.4)	
Left	67 (42.9)	23.0 (16.0–36.0)		16 (41.0)	51 (43.6)	
Tumor thrombus level			0.002			0.010
RV	85 (54.5)	20.0 (15.5–32.0)		12 (30.8)	73 (62.4)	
IVC	71 (45.5)	27.0 (20.0–45.0)		27 (69.2)	44 (37.6)	
Maximum tumor width, cm			0.421			0.502
>10	57 (36.5)	24.0 (17.0–37.0)		16 (41.0)	41 (35.0)	
<10	99 (63.5)	22.0 (15.0–42.0)		23 (59.0)	76 (65.0)	
Pathological stage			0.004			0.001
T3a	80 (51.3)	20.0 (15.25–32.0)		11 (28.2)	69 (59.0)	
T3b-4	76 (48.7)	26.0 (18.3–44.8)		28 (71.8)	48 (41.0)	
Lymph node metastasis			0.814			0.480
Yes	19 (12.2)	23.0 (15.0–38.0)		6 (15.4)	13 (11.1)	
No	137 (87.8)	23.0 (16.5–37.0)		33 (84.6)	104 (88.9)	
Fuhrman grade			0.175			0.011
G1 + G2	91 (58.3)	23.0 (16.0–35.0)		16 (41.0)	75 (65.1)	
G3 + G4	65 (41.7)	23.0 (17.0–46.0)		23 (59.0)	42 (35.9)	
Histological subtypes			0.714			0.881
Clear-cell RCC	139 (89.1)	23.0 (16.0–38.0)		35 (89.7)	104 (88.9)	
Non-clear-cell RCC	17 (10.9)	27.0 (16.5–39.0)		4 (10.3)	13 (11.1)	
Sarcomatoid feature			0.114			0.010
Yes	27 (17.3)	28.0 (17.0–54.0)		12 (30.8)	15 (12.8)	
No	129 (82.7)	23.0 (16.0–36.0)		27 (69.2)	102 (87.2)	

GGT: gamma-glutamyltransferase; IQR: interquartile range; BMI: body mass index; ASA: American Society of Anesthesiologists; RV: renal vein; IVC: inferior vena cava; RCC: renal cell carcinoma.

**Table 2:** Univariate analysis of various variables for predicting CSS and RFS.

Variables	CSS		RFS	
	HR (95% CI)	*p* value	HR (95% CI)	*p* value
Age, year (≥60 versus <60)	1.315 (0.731–2.367)	0.361	1.204 (0.743–1.951)	0.451
BMI, kg/m^2^ (<25 versus ≥25)	1.769 (0.853–3.669)	0.125	2.387 (0.733–2.260)	0.379
Gender (female versus male)	1.457 (0.799–2.659)	0.220	1.042 (0.611–1.777)	0.879
Symptom presentation (yes versus no)	2.430 (1.172–5.036)	0.017	1.546 (0.899–2.657)	0.115
ASA (3 + 4 versus 1 + 2)	1.488 (0.691–3.203)	0.310	1.674 (0.892–3.140)	0.109
Tumor laterality (right versus left)	1.189 (0.890–1.588)	0.241	1.052 (0.826–1.339)	0.682
Tumor thrombus level (IVC versus RV)	1.531 (1.130–2.074)	0.006	1.345 (1.055–1.715)	0.017
Maximum tumor width, cm (≥10 versus <10)	1.392 (1.041–1.860)	0.026	1.258 (0.987–1.603)	0.064
Pathological stage (T3b-4 versus T3a)	2.926 (1.539–5.563)	<0.001	2.170 (1.315–3.578)	0.002
Lymph node invasion (N+ versus N0/Nx)	1.885 (0.909–3.909)	0.088	1.802 (0.963–3.370)	0.065
Fuhrman grade (G3 + G4 versus G1 + G2)	2.306 (1.275–4.173)	0.006	2.024 (1.247–3.285)	0.004
Histological subtype (clear-cell versus non-clear-cell)	1.038 (0.652–1.653)	0.875	1.057 (0.714–1.565)	0.781
Sarcomatoid feature (yes versus no)	1.740 (0.901–3.364)	0.099	1.692 (0.976–2.936)	0.061
GGT, IU/L (≥37.5 versus <37.5)	2.992 (1.668–5.368)	<0.001	2.587 (1.580–4.236)	<0.001

CSS: cancer-specific survival; RFS: recurrence-free survival; HR: hazard ratio; BMI: body mass index; ASA: American Society of Anesthesiologists; IVC: inferior vena cava; RV: renal vein; GGT: gamma-glutamyltransferase.

**Table 3:** Multivariate Cox regression model analysis of predictive factors of CSS and RFS.

Variables	CSS		RFS	
	HR (95% CI)	*p* value	HR (95% CI)	*p* value
Symptom presentation (yes versus no)	1.648 (0.774–3.509)	0.195	—	—
Maximum tumor width, cm (≥10 versus <10)	2.000 (1.116–3.583)	0.020	—	—
Pathological stage (T3b-4 versus T3a)	2.274 (1.179–4.385)	0.014	1.808 (1.079–3.028)	0.024
Fuhrman grade (G3 + G4 versus G1 + G2)	1.860 (1.007–3.434)	0.047	1.724 (1.051–2.827)	0.031
GGT, IU/L (≥37.5 versus <37.5)	2.115 (1.164–3.843)	0.014	1.955 (1.166–3.276)	0.011

CSS: cancer-specific survival; RFS: recurrence-free survival; HR: hazard ratio; GGT: gamma-glutamyltransferase.
